# Mammary Fat of Breast Cancer: Gene Expression Profiling and Functional Characterization

**DOI:** 10.1371/journal.pone.0109742

**Published:** 2014-10-07

**Authors:** Fengliang Wang, Sheng Gao, Fei Chen, Ziyi Fu, Hong Yin, Xun Lu, Jing Yu, Cheng Lu

**Affiliations:** 1 Department of Breast Surgery, Nanjing Maternity and Child Health Care Hospital Affiliated to Nanjing Medical University, Nanjing, People’s Republic of China; 2 Jinling High School, Nanjing, People’s Republic of China; 3 Department of Geratology, the First Hospital Affiliated to Nanjing Medical University, Nanjing, People’s Republic of China; University of North Carolina School of Medicine, United States of America

## Abstract

Mammary fat is the main composition of breast, and is the most probable candidate to affect tumor behavior because the fat produces hormones, growth factors and adipokines, a heterogeneous group of signaling molecules. Gene expression profiling and functional characterization of mammary fat in Chinese women has not been reported. Thus, we collected the mammary fat tissues adjacent to breast tumors from 60 subjects, among which 30 subjects had breast cancer and 30 had benign lesions. We isolated and cultured the stromal vascular cell fraction from mammary fat. The expression of genes related to adipose function (including adipogenesis and secretion) was detected at both the tissue and the cellular level. We also studied mammary fat browning. The results indicated that fat tissue close to malignant and benign lesions exhibited distinctive gene expression profiles and functional characteristics. Although the mammary fat of breast tumors atrophied, it secreted tumor growth stimulatory factors. Browning of mammary fat was observed and browning activity of fat close to malignant breast tumors was greater than that close to benign lesions. Understanding the diversity between these two fat depots may possibly help us improve our understanding of breast cancer pathogenesis and find the key to unlock new anticancer therapies.

## Introduction

The fully differentiated breast is composed of two cell compartments. The gland, composed of branched ducts, constitutes the epithelial compartment and lobuloalveolar differentiated units that are able to produce milk proteins when necessary. This gland is embedded in the connective tissue compartment commonly referred to as the mammary fat pad because it mainly contains adipose tissue [1]. Breast cancer is thought to occur initially in mammary epithelial cells [2], [3]. Any discussion of processes involving mammary glandular growth, differentiation, lactation or involution is generally centered on changes within the epithelial tissue [4]–[6]. However, a unique and fascinating aspect of mammary gland biology is the requirement for mammary epithelial cells to grow and function in the mammary fat pad [7].

The mammary fat pad is also named stroma. Stroma is composed of an extracellular matrix and a variety of cell types including endothelial cells, inflammatory cells, fibroblasts, fibroblast-like cells and adipocytes. Dynamic and reciprocal communication between epithelial and stromal compartments occurs during breast cancer progression [8], [9]. Stromal cells generally promote tumor progression by secreting chemokines, growth factors and extracellular matrix components [9]. Understanding the alterations in the tissue architecture accompanying the development of breast cancer is likely to improve the early detection of breast cancer and ultimately, the treatment of patients. To date, most of the studies focusing on cancer cell-stromal cell interactions, have usually emphasized the contribution of fibroblasts, inflammatory and endothelial cells [8], [10]. Nonetheless, rare attention has been given to mammary fat since this depot of adipose tissue was once viewed as a relatively inert matrix.

Compositionally, adipose tissue is the major contributor to the volume of the breast. Two types of adipose tissue: white adipose tissue (WAT) and brown adipose tissue (BAT) have been distinguished histologically and functionally [11], [12]. WAT is composed of cells with a large lipid droplet that is stored and used as fuel. In contrast, BAT is composed of cells with numerous small lipid droplets to facilitate the catabolism of lipids for heat production. There is an increasing amount of evidence that mammals have the ability to expand the number and activity of “brown adipocytes” within white fat depots, termed the browning of white fat [13], [14]. It has been verified that expression of uncoupling protein 1 (UCP1), the main functional factor of brown adipose, regulates the growth of aggressive human tumors such as skin carcinoma and colon cancer [15], [16]. However, insights into UCP1 expression and the role of mammary fat browning in breast cancer are rather limited.

In the present study, we analyzed gene expression profiling and functionally characterized mammary fat from 60 Chinese women, among which 30 subjects had breast cancer and 30 that had benign breast lesions. Adipogenesis-related genes and secretion-related genes were detected. Most importantly, this study focused on the browning of white adipose tissue, with the aim of finding specific roles of mammary fat in the occurrence and progression of breast cancer.

## Materials and Methods

### Subjects and samples

Samples of mammary fat were obtained from 60 Chinese women who underwent lumpectomy. 30 subjects had breast cancer and the others had benign breast lesions. All subjects had stable weight with fluctuations of not more than 2% of their body weight for at least three months prior to surgery. Patients fasted for at least 8 h and were not treated with any hormones that might influence metabolism prior to the acquisition of samples. None of the patients had ever received medication known to influence adipose function or metabolism. Fat tissue samples obtained were adjacent to the tumor mass and were devoid of fibrosis, as assessed macroscopically. Immediately after excision, adipose tissue samples (10–20 g) were transported to the laboratory.

The Ethics Committee of Nanjing Medical University approved this study. Individuals in this study gave written informed consent to publish their case details.

### Human adipose stromal vascular fraction (SVF) cell culture and differentiation

Human SVF cells were isolated from mammary fat tissues of subjects, as described previously [17]. Harvested fat was washed in 0.1% BSA-PBS, finely minced, and digested in 2 mg/mL type I collagenase solution (Gibco, Gaithersburg, USA) for 40 min at 37°C with vigorous shaking. The resulting cell suspension was then filtered through a 150-µm nylon mesh, and adipocytes were removed using centrifugation and washed with 0.1% BSA-PBS. Fresh SVF cells were seeded into plates (5×10^4^ cells/cm^2^) in fresh growth medium (DMEM/Ham’s F-12 medium with 10% fetal bovine serum, 100 U/mL penicillin and 100 mg/mL streptomycin) (Sigma-Aldrich, St. Louis, USA) and allowed to adhere to the culture dish overnight. The next day, cells were washed to remove floating cells and cultured in growth medium for 5–7 days until the cells reached confluence (Day 0).

For differentiation experiments, cells were grown in a serum-free differentiation medium after they had reached confluence (DMEM/Ham’s F-12 medium containing 15 mM NaHCO_3_, 15 mM HEPES, 33 mM biotin, 17 mM pantothenate, 0.2 nM triiodothyronine, 66 nM human insulin and10^−6^ M dexamethasone) (Sigma-Aldrich, St. Louis, USA). To induce maximal differentiation, confluent cells were supplemented with differentiation medium containing 0.5 mM 3-isobutyl-1-methylxanthine (Sigma-Aldrich, St. Louis, USA) during the first 3 days. After 14 days in differentiation medium, most cells were induced into mature adipocytes (Day 14). Differentiated adipocytes were harvested for further study.

### Oil red O staining

After the induction of differentiation, cells were washed with PBS and fixed with 10% formalin in PBS for 1 hour, washed 3 times with water and finally air-dried. Cells were stained with oil red O (Sigma-Aldrich, St. Louis, USA) (six parts of saturated oil red O dye [0.6%] in isopropanol and four parts of water) for 30 min. Excess stain was removed by washing with 70% ethanol. Stained cells were then washed with water and stored in PBS for visualization under a phase-contrast microscope (Olympus, Tokyo, Japan). Quantitative determination of triglycerides was obtained by measuring the absorbance of an extraction of the stained lipid at 510 nm in a spectrophotometer (Amersham Biosciences, Reno, USA).

### Mammary fat tissue histology and immunohistochemistry

Mammary fat tissues were fixed in 10% formalin, processed and paraffin-embedded. Multiple sections (5 µm) were prepared and stained with hematoxylin and eosin for general morphological observation. For immunocytochemical staining, sections of fat tissues were incubated with anti-UCP1 antibodies (1∶1000, Abcam, MA, USA) for 30 min at room temperature. Signals were detected using a biotinylated goat anti-rabbit secondary antibody (Vector Laboratories, CA, USA) in combination with the ABC kit (Vector Laboratories, CA, USA) and DAB substrate (Vector Laboratories, CA, USA). Samples were visualized using a Nikon Eclipse 80i upright microscope (Nikon, Tokyo, Japan). The intensity of the staining signal was measured and documented using Image-Pro Plus 6.0 image analysis software (Media Cybernetics, Inc. Silver Spring, MD, USA). The mean densitometry of the digital image was designated as representative UCP1 staining intensity (indicating relative UCP1 expression). The signal density of tissue areas from five randomly selected images were counted in a blinded manner and subjected to statistical analysis.

### Quantitative real-time PCR

Total RNA was isolated from tissues or cells with the TRIzol reagent (Invitrogen, CA, USA), according to the manufacturer’s instructions. A sample of total RNA (2 µg) was reverse-transcribed with 200 U Moloney murine leukemia virus reverse transcriptase (M-MLV, Promega, Madison, WI, USA) in the presence of 0.5 mmol/L deoxynucleotide triphosphate, 25 U RNase inhibitor and 10 µM random hexamer primers, in a total volume of 25 µL. PCR primers were designed using Primer5 software (Table S1). Each quantitative real-time PCR was carried out in triplicate, in a 25 µL volume of SYBR Green Real-time PCR Master Mix (Roche, Basel, Switzerland). PCR conditions were as follows: 10 min at 95°C, followed by 40 cycles of 15 s at 95°C and 60 s at 60°C. The temperature was heated from 60°C to 95°C and the PCR melting curve was made every 1.0°C after the amplification reaction (ABI 7000, StepOnePlus, CA, USA).

The mean value of triplicates for each sample was calculated and expressed as the cycle threshold (Ct). For each gene studied, the amount of gene expression was then calculated as the ΔCt, the difference between the Ct value of the sample and the Ct value of GAPDH, which was used as an endogenous control. The relative expression level was evaluated using the comparative delta-delta Ct method [18]. Expression for each gene in fat from benign lesions was arbitrarily set at 1 to facilitate comparison between negative control fat (NCF) and cancer-associated fat (CAF).

### Protein extraction and western blotting

Mammary fat tissues were washed twice with ice-cold PBS and ground using a refiner. Total tissue protein was extracted with RIPA buffer (50 mM Tris-HCl, pH 7.4, 150 mM NaCl, 1% (v/v) Nonidet-P40, 1 mM EDTA, 1 mM NaF, 10 µg/mL aprotinin, 10 µM leupeptin, and 1 mM phenylmethanesulfonyl fluoride (PMSF), Amresco, PA, USA) and allowed to stand on ice for 30 min to permit lysis. After centrifugation at 11,000 rpm for 10 min at 4°C, protein content in the supernatant was collected and the protein concentration was determined using a BCA assay (Pierce, Rockford, USA). Aliquots (60 µg) of protein were separated on a 12% SDS-polyacrylamide gel electrophoresis and transferred onto a poly (vinylidene difluoride) membrane (Millipore, Boston, USA). The membrane was blocked with 1% (w/v) BSA (Amresco, PA, USA) in TBST (10 mM Tris-HCl, pH 7.8, 150 mM NaCl and 0.1% Tween-20) for 2 h and then incubated with a goat anti-human FABP4 primary antibody (1∶200, Santa Cruze, CA, USA), a rabbit anti-human HSL primary antibody (1∶200, Santa Cruze, CA, USA) or a rabbit anti-human UCP1 primary antibody (1∶1000, Abcam, MA, USA) in TBST containing 1% (w/v) BSA overnight at 4°C. Blots were treated with horseradish peroxidase-conjugated anti-goat and anti-rabbit IgG (1∶10,000, KPL, Maryland, USA) in TBST containing 1% (w/v) BSA for 60 min, and immune complexes were detected using an ECL plus detection kit (Cell Signaling Technology, MA, USA). Bands were quantified using densitometric image analysis software (Quantity One, Bio-Rad, CA, USA). The relative expression of FABP4, HSL and UCP1 were normalized to that of GAPDH.

### Statistical analysis

Statistical analysis was performed using SPSS 18.0 software. Results were presented as means ± SEM. Statistically significant differences were calculated using a Student’s *t*-test. A *p*-value of <0.05 was considered significant.

## Results

### Subject characterization

60 patients were studied: 30 subjects with breast cancer and 30 subjects with benign breast lesions. Of the 60 subjects, 26 were premenopausal and 34 were postmenopausal at the time of disease diagnosis. The age of the patients ranged from 35 to 60, with a mean age of 49 years. Tumor size, lymph node metastasis, TNM stage and molecular tumor type were shown in Table 1. The pathology of all 30 subjects with breast cancer showed that they were all invasive ductal carcinomas. Benign breast lesions were diagnosed as either breast adenomas or adenosis of the breast. Anthropometric and metabolic characteristics of these subjects are shown in Table 2.

**Table 1:** Participant characteristics.

	Breast tumor (n = 30)	Benign breast lesion (n = 30)	
**Age**			
≤45	10	10	*p*>0.05
45–55	10	10	
>55	10	10	
**Menstrual status**			
Premenopausal	12	14	*p*>0.05
Postmenopausal	18	16	
**Tumor size(cm)**			
≤2	14	13	*p*>0.05
>2	16	17	
**Lymphnode metastasis**			
Positive	8		
Negative	22		
**TNM stage**			
I	14		
II	13		
III	3		
**Molecular tumor type**			
Luminal A	10		
Luminal B	10		
Base-like	7		
HER2-positive	3		

**Table 2:** Anthropometric and metabolic characteristics of the study groups.

	Breast tumor (n = 30)	Benign breast lesion (n = 30)	*p*-value
	Mean ± SEM	Mean ± SEM	
BMI (kg/m^2^)	23.5±0.4	22.6±1.3	>0.05
WHR	0.89±0.02	0.85±0.01	>0.05
Fasting plasma glucose (mmol/l)	5.0±0.3	4.8±0.4	>0.05
Systolic blood pressure (mmHg)	127±9.7	126±8.9	>0.05
Diastolic blood pressure (mmHg)	75±5.7	80±13.6	>0.05
Total cholesterol (mmol/l)	3.60±0.63	4.11±0.49	>0.05
HDL cholesterol (mmol/l)	1.13±0.27	1.25±0.27	>0.05
LDL cholesterol (mmol/l)	2.05±0.42	2.14±0.66	>0.05
TG (triglyceride) (mmol/l)	1.19±0.40	1.23±0.71	>0.05

### Gene expression profiling and functional characterization of mammary fat

**Figure 1:**
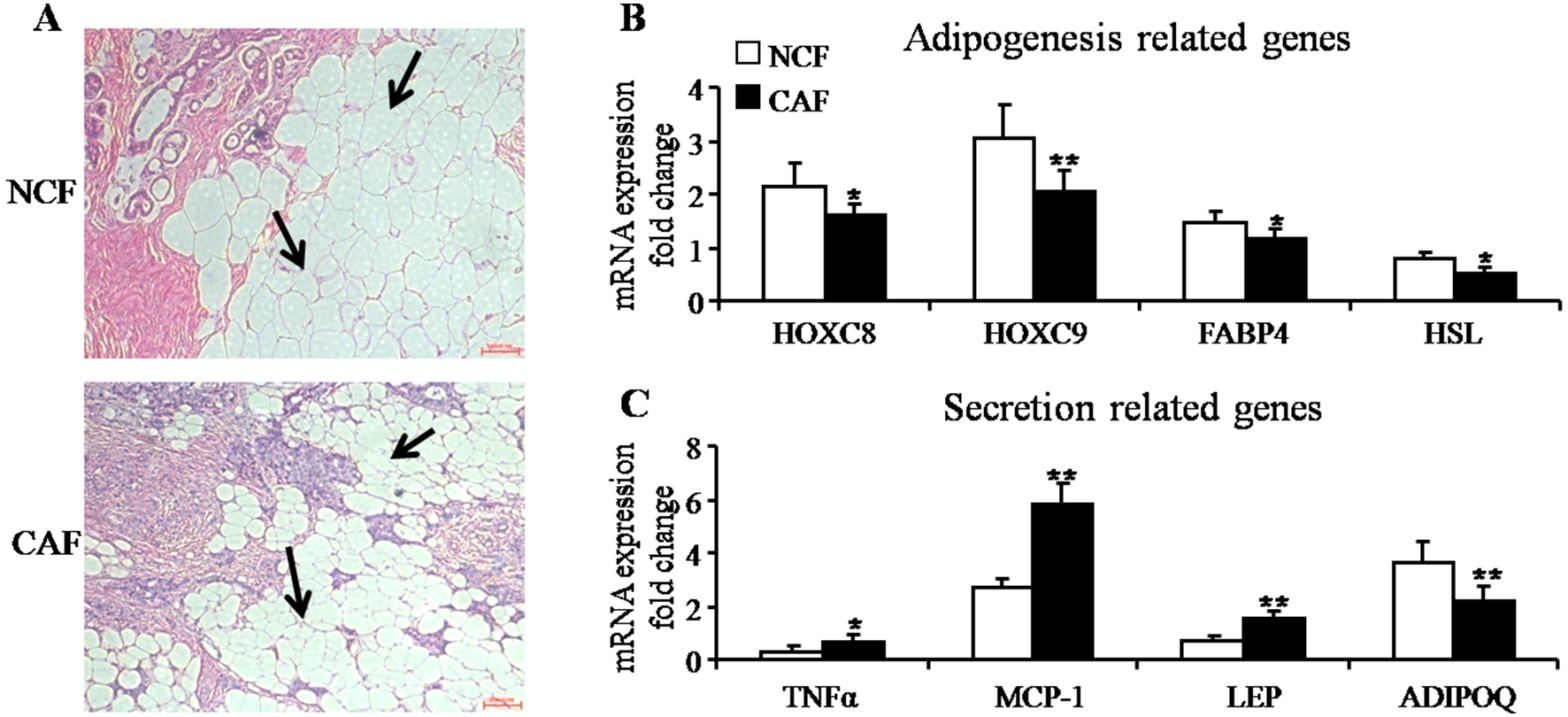
Adipogenesis and secretion-related gene expression of mammary fat tissues. (A) Representative images of H&E-stained sections of mammary fat from one patient with breast cancer (cancer associated fat, CAF) and from one with a benign lesion (negative control fat, NCF). Black arrows point to adipocytes (magnification100×). (B) Quantitative RT-PCR analysis of mRNA expression of adipogenesis-related genes: HOXC8, HOXC9, FABP4 and HSL (mean± SEM; n = 30; *P<0.05, **P<0.01). (C) Quantitative RT-PCR analysis of mRNA expression of secretion-related genes: TNFα, MCP-1, LEP and ADIPOQ (mean ± SEM; n = 30; *P<0.05, **P<0.01).

To investigate the distinctive features of mammary fat, we collected fat tissue close to the tumors from breast cancer (CAF) and benign breast lesions (NCF). Histological images of adipose tissue adjacent to breast tumors contained fewer and smaller adipocytes compared with the fat adjacent to benign lesions (Figure 1A). We then studied the mRNA expression of adipogenesis-related genes: HOXC8 (homeobox genes family C8), HOXC9 (homeobox genes family C9), FABP4 (fatty acid binding protein 4) and HSL (hormone-sensitive lipase). mRNA levels of these genes were up-regulated in the CAF group (Figure 1B). Moreover, CAF had abnormal levels of inflammation-related factors, such as TNFα (tumor necrosis factor alpha) and MCP-1 (monocyte chemotactic protein 1) (Figure 1C). Furthermore, secretion function-related genes, ADIPOQ (adiponectin) and LEP (leptin) were detected. As shown in Figure 1C, the expression of ADIPOQ was down-regulated, whereas that of LEP was up-regulated. These results suggested that fat tissue adjacent to malignant and benign lesions exhibited distinct gene expression profiles and functional characteristics. Although the mammary fat of breast tumors atrophied, they secreted an increased level of tumor growth stimulatory factors.

### Indications for fat browning activity of mammary fat

**Figure 2:**
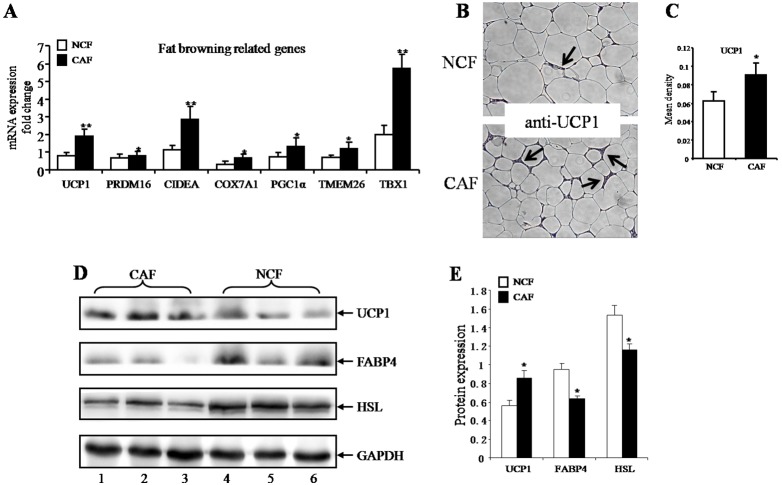
Browning of mammary fat from breast tumors and benign lesions. (A) Quantitative RT-PCR analysis of mRNA expression of fat browning-related genes: UCP1, PRDM16, CIDEA, COX7A1, PGC1α, TMEM26 and TBX1 (mean±SEM; n = 30; *P<0.05, **P<0.01). (B) Immunohistochemically stained UCP1 sections of mammary fat from breast tumors (cancer associated fat, CAF) and from benign lesions (negative control fat, NCF). Black arrows point to UCP1 staining (magnification100×). (C) The mean densitometry of the digital image is designated as representative UCP1 staining intensity (indicating the relative UCP1 expression level). The mean density indicating the relative UCP1 expression level was higher in the CAF group (mean ± SEM; n = 6; *P<0.05). (D) Western blotting of mammary fat tissue protein from breast tumors and benign lesions. Lanes 1–3 from CAF and lanes 4–6 from NCF. (E) Bands were quantified using densitometric image analysis software. The relative expression of FABP4, HSL and UCP1 were normalized to that of GAPDH (mean ± SEM; n = 3; *P<0.05).

To assess the browning activity of fat from mammary fat pads, we evaluated expression levels of marker genes using quantitative real-time PCR and western blotting. Expression of UCP1 (uncoupling protein 1), PRDM16 (PR domain-containing 16), CIDEA (cell death-inducing DFFA-like effectorprotein A), COX7A1 (cytochrome c oxidase subunit VIIa polypeptide 1), PGC1α (peroxisome proliferator-activated receptor gamma coactivator 1a), TMEM26 (transmembrane protein 26) and TBX1 (T-box 1) all increased in CAF when compared with NCF (Figure 2A). Immunohistochemically stained UCP1 sections of mammary fat are shown in Figure 2B. The mean densitometry of the digital image was designated as representative UCP1 staining intensity. The mean density, indicating the relative UCP1 expression level, was higher in the CAF group (Figure 2C). The protein level of UCP1 also increased in the CAF group (Figure 2D). We concurrently detected protein levels of FABP4 and HSL, and the results were consistent with mRNA expression (Figure 2D). Bands were quantified using densitometric image analysis software (Figure 2E). These results indicated that mammary stroma contains brown adipose tissue and greater numbers of these cells are present close to breast cancers as compared with the tissue surrounding benign lesions.

### The morphology and gene expression of adipocytes from mammary fat

**Figure 3:**
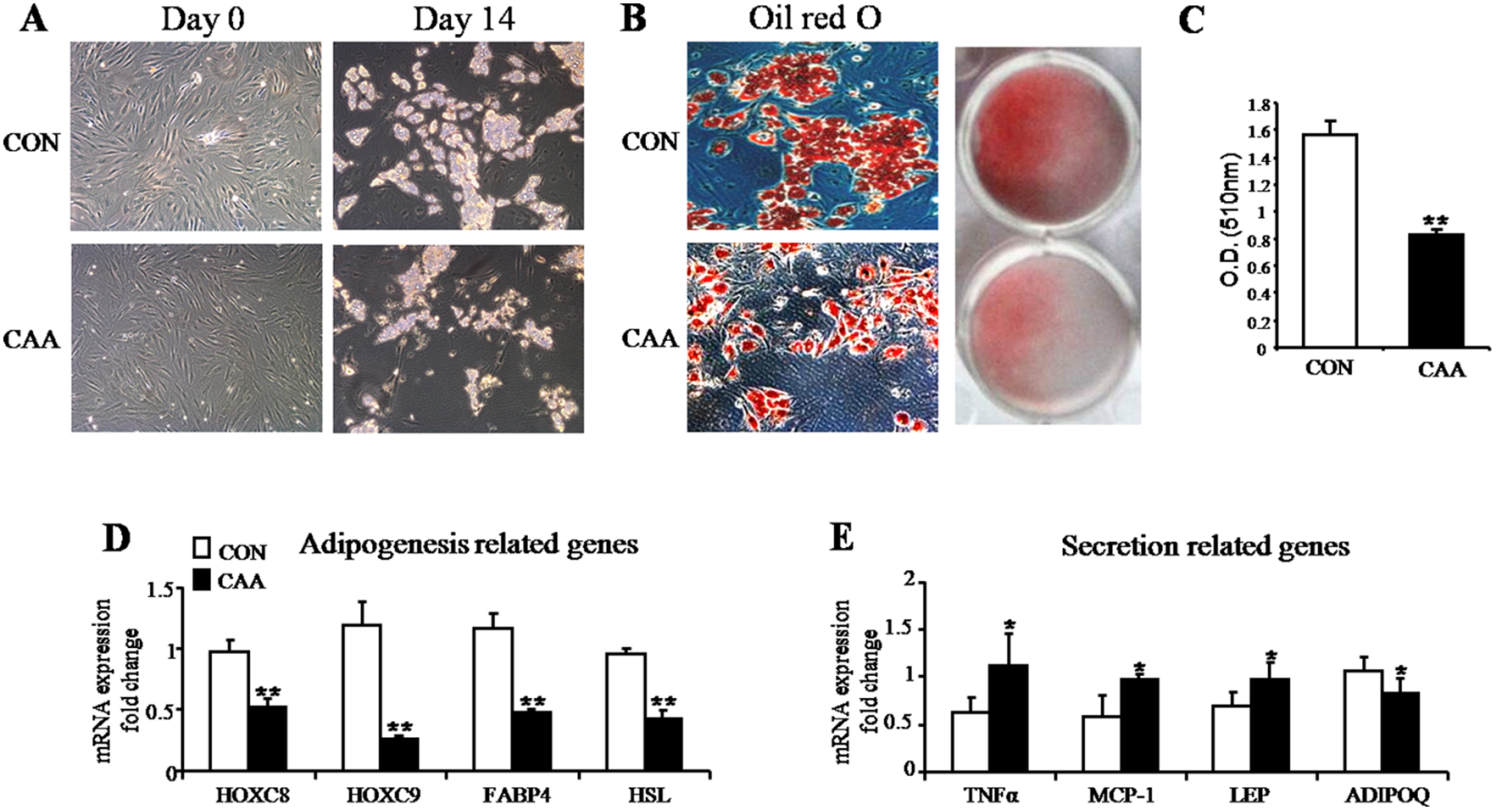
Adipogenesis and secretion function-related gene expression in SVF cells from mammary fat. (A) Representative images of the human adipose SVF cells harvested from mammary fat of breast tumors (cancer associated adipocytes, CAA, lower slides) and benign lesions (control adipocytes, CON, upper slides). Day 0 = preadipocytes, Day 14 = mature adipocytes (magnification100×). (B) Cellular lipid accumulation stained with oil red O imaged through a phase contrast microscope. Lower slides, magnification 100×. (C) Quantitative determination of triglycerides by measuring the absorbance of an extraction of the stained lipid at 510 nm in a spectrophotometer. Accumulation of lipid droplets increased in mature CAA (mean ± SEM; n = 6; *P<0.05). (D) Quantitative RT-PCR analysis of mRNA expression of adipogenesis-related genes: HOXC8, HOXC9, FABP4 and HSL (mean ± SEM; n = 6; *P<0.05, **P<0.01). (E) Quantitative RT-PCR analysis mRNA expression of secretion-related genes: TNFα, MCP-1, LEP and ADIPOQ (mean ± SEM; n = 6; *P<0.05, **P<0.01).

To further clarify the differentiation features of adipocytes from mammary fat tissues, we primarily cultured human adipose SVF cells and then induced maturation. The number of preadipocytes harvested from the same amount of fat tissue was reduced in CAA (cancer-associated adipocytes) as compared to in CON (control adipocytes from benign lesions). At Day 14, the lipid drops in CAA was also less than that in adipocytes from benign breast lesions (Figure 3A). Accumulation of lipid droplets increased in mature CAA (Figure 3B–C). mRNA expression levels of adipogenesis-related genes HOXC8, HOXC9, FABP4 and HSL were down-regulated in CAA (Figure 3D). Expression of inflammation-related genes, TNFα and MCP-1, increased (Figure 3E). The level of adipokines (e.g. leptin) also increased but adiponectin was down-regulated (Figure 3E). The ability of preadipocytes from the mammary fat of breast tumors to differentiate was decreased compared with that of preadipocytes from benign lesions.

### The fat browning activity of adipocytes from mammary fat

**Figure 4:**
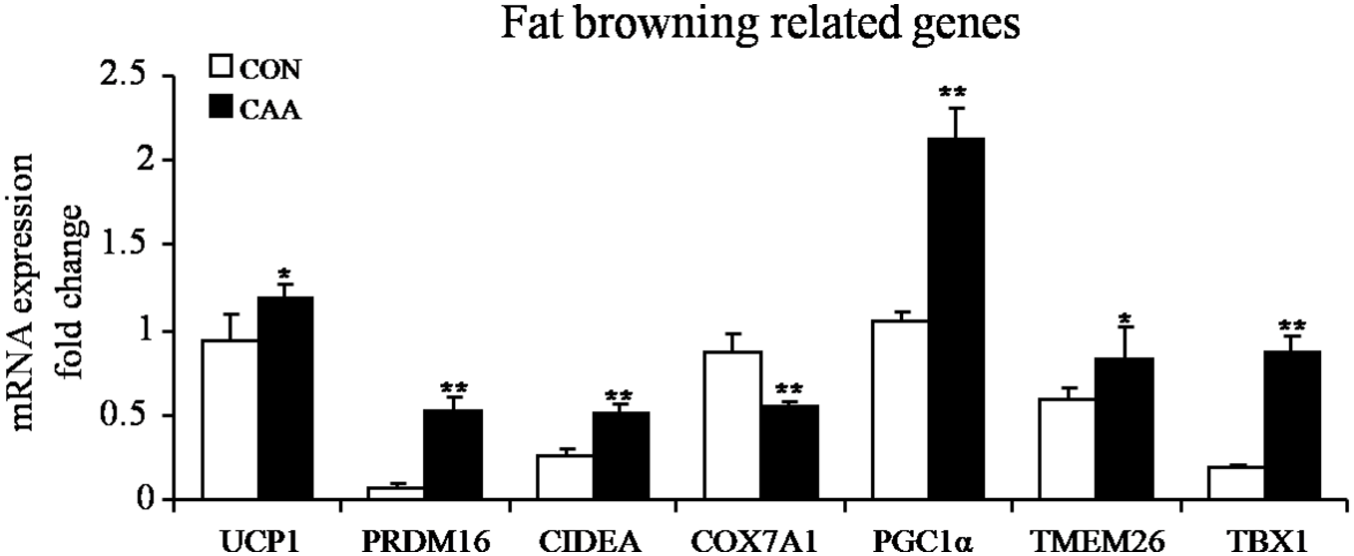
Browning of mammary fat cells from breast tumors and benign lesions. Human adipose SVF cells were harvested from the mammary fat of breast tumors and benign lesions. SVF cells were then cultured and induced to mature (Day 14). Quantitative RT-PCR analysis of mRNA expression of fat browning-related genes: UCP1, PRDM16, CIDEA, COX7A1, PGC1α, TMEM26 and TBX1 (mean ± SEM; n = 6; *P<0.05, **P<0.01). (Cancer associated adipocytes, CAA; control adipocytes, CON).

To further study the browning activity of adipocytes from mammary fat, SVF cells were cultured and induced to mature. Marker gene expression was detected using quantitative real-time PCR. This included the up-regulation of UCP1, PRDM16, CIDEA, COX7A1, PGC1α, TMEM26 and TBX1 expression (Figure 4) in CAA. Thus, to a great extent, the mRNA expression profile observed in tissues was largely conserved in isolated adipocytes.

## Discussion

Several studies have demonstrated that cancer is a tissue-based disease in which malignant cells interact dynamically with multiple normal cell types such as fibroblasts, infiltrating inflammatory cells, endothelial cells and adipocytes, within the context of the extracellular matrix [8]. Adipocytes are less well studied compared with other cell types present in the microenvironment, despite the fact that they are one of the most prominent cell types in some tissues such as breast [19]. The mammary gland is a dynamic organ that continually changes its architecture and function. Reciprocal interactions between the epithelium and connective tissue exert profound effects on mammary gland morphogenesis, development and homeostasis, even though the details of these events are not fully understood [20], [21].

Until recently, adipocytes were mainly considered as an energy storage depot, whereas there is clear evidence indicating that adipocytes can act as endocrine cells producing hormones, growth factors and adipokines [22]. Adipocytes participate in a highly complex vicious cycle orchestrated by cancer cells to promote tumor invasion [23]. Meanwhile, invasive cancer cells dramatically impact surrounding adipocytes that exhibit an altered phenotype and specific biological features (e.g. delipidation and overexpression of inflammatory cytokines) [23]. These observations are in agreement with our results in this study. We demonstrated that the expression of adipogenesis-related genes HOXC8, HOXC9, FABP4 and HSL in adipose tissue adjacent to malignant breast tumors was down-regulated and the level of inflammatory cytokines, like TNFα and MCP-1, was up-regulated.

There is increasing evidence that adiponectin and leptin, secreted by peritumoral adipose tissue in several cancers including breast, are important [24], [25]. Leptin appears to be a positive factor for tumor development and aggressiveness, while adiponectin protects against cancers. Ruben *et al.* confirmed that pleiotropic effects of leptin in breast cancer involved the enhancement of cell proliferation and pro-angiogenic actions linked to leptin-induced expression of cell cycle proteins and regulators in addition to anti-apoptotic and inflammatory factors [26]. We also found that the level of leptin in breast cancer-associated adipose tissue increased while adiponectin decreased. While adipogenesis decreased in mammary fat of breast tumors, the fat cells continued to secrete factors that stimulate tumor progression.

Stephen *et al.* demonstrated that the mammary gland contained brown adipose tissue and played a potential role in adaptive thermogenesis [27]. However, few studies have thus far reported on the browning ability of mammary fat. Rosa *et al.* recently showed that highly expressed UCP1 in cancer-associated fibroblasts significantly promoted tumor growth *via* the generation of high-energy mitochondrial fuels (such as ketone bodies) [28]. It has also been found that in pancreatic cells, expression of UCP1 increased oxygen consumption and decreased tumor growth [29]. In our research, expression of marker genes of fat browning, like UCP1, PRDM16, CIDEA, COX7A1, PGC1α, TMEM26 and TBX1, was up-regulated in adipose tissue adjacent to breast tumors. Expression of these genes may help limit tumor growth, given that fat browning is related to mitochondrial function. In such a situation, understanding the specific role of fat browning and mitochondrial dysfunction in cancer pathogenesis may be the key to unlocking new anticancer therapies.

In conclusion, our data indicated that gene expression and functional characteristics were different in mammary fat from breast tumors as compared to benign lesions. Probing the diversity between these two fat depots will help us improve our understanding of breast cancer pathogenesis.

## Supporting Information

Table S1 **PCR Oligonucleotide Primers and Annealing Temperature.**
(DOCX)
