# Supplementary material for: Mammary Fat of Breast Cancer: Gene Expression Profiling and Functional Characterization
Source: PLoS One. 2014 Oct 7;9(10):e109742. doi: 10.1371/journal.pone.0109742 (PMC4188628; doi:10.1371/journal.pone.0109742)
Supplement: Table S1 — PCR Oligonucleotide Primers and Annealing Temperature. (DOCX) [file pone.0109742.s001.docx]

| Table S1. PCR Oligonucleotide Primers and Annealing Temperature. | | |
| --- | --- | --- |
| **Gene**  **(Accession)** | **Primers(5’-3’)** | **Annealing**  **Temperature (°C)** |
| HOXC8  (NM_022658) | FW: AACTCAGGCTACCAGCAGAAC  RV:TGAGCCCCATAAAGGGACTGT | 60 |
| HOXC9  (NM_006897) | FW: ACTCGCTCATCTCTCACGACA  RV:GACGGAAAATCGCTACAGTCC | 60 |
| FABP4  (NM_001442) | FW: ACACCGAGATTTCCTTCAAACTG  RV: CCATCTAGGGTTATGATGCTCTTCA | 60 |
| HSL  (NM_005357) | FW: CCTCATGGCTCAACTCC  RV: GGTTCTTGACTATGGGTGA | 60 |
| TNFα  (NM_000594) | FW: CCCCAGGGACCTCTCTCTAAT  RV:GGTTTGCTACAACATGGGCTAC | 60 |
| MCP1  (NM_002982) | FW: TGTCCCAAAGAAGCTGTGATCT  RV:GGAATCCTGAACCCACTTCTG | 60 |
| LEP  (NM_000230) | FW: CACCAAAACCCTCATCAAGACA  RV:CTTTCTGTTTGGAGGAGACTGACT | 60 |
| ADIPQ  (NM_001177800) | FW: CGTGATGGCAGAGATGGC  RV:CCGGTTTCACCGATGTCT | 60 |
| UCP1  (NM_021833) | FW: CACCTTCCCGCTGGACACT  RV:CCCTAGGACACCTTTATACCTAATGG | 60 |
| PRDM16  (NM_022114) | FW: CGAGGCCCCTGTCTACATTC  RV:GCTCCCATCCGAAGTCTGTC | 60 |
| CIDEA  (NM_001279) | FW: GATGCCCTCGTCATCGCTAC  RV:GCGTGTTGTCTCCCAAGGTC | 60 |
| COX7A1  (NM_001864) | FW: CCGCTTTCAGAACCGAGTG  RV:CCCTTCAGGTACAACGGGA | 60 |
| PGC1α  (NM_013261) | FW: TCTGAGTCTGTATGGAGTGACAT  RV:CCAAGTCGTTCACATCTAGTTCA | 60 |
| TMEM26  (NM_178505) | FW: TGAGACGGCCAAAGTTTTTGT  RV:TGGGTAGAAGCCATCTTCCAA | 60 |
| TBX1  (NM_005992) | FW: TAGCGAGAAATATGCCGAGGA  RV:CGTGATCCGATGGTTCTGGT | 60 |
| GAPDH  (NM_001256799) | FW: ACAACTTTGGTATCGTGGAAGG  RV: GCCATCACGCCACAGTTTC | 60 |
